# Comparison of 2D, 2.5D, and 3D segmentation networks for maxillary sinuses and lesions in CBCT images

**DOI:** 10.1186/s12903-023-03607-6

**Published:** 2023-11-15

**Authors:** Yeon-Sun Yoo, DaEl Kim, Su Yang, Se-Ryong Kang, Jo-Eun Kim, Kyung-Hoe Huh, Sam-Sun Lee, Min-Suk Heo, Won-Jin Yi

**Affiliations:** 1 Department of Oral and Maxillofacial Radiology and Dental Research Institute, School of Dentistry, Seoul National University, Seoul, Korea; 2 Interdisciplinary Program in Bioengineering, Graduate School of Engineering, Seoul National University, Seoul, Korea; 3 Department of Applied Bioengineering, Graduate School of Convergence Science and Technology, Seoul National University, Seoul, Korea; 4 Department of Biomedical Radiation Sciences, Graduate School of Convergence Science and Technology, Seoul National University, Seoul, Korea

**Keywords:** Deep learning, CBCT image, Maxillary sinus segmentation, Maxillary sinus lesion segmentation, 2.5D network

## Abstract

**Background:**

The purpose of this study was to compare the segmentation performances of the 2D, 2.5D, and 3D networks for maxillary sinuses (MSs) and lesions inside the maxillary sinus (MSL) with variations in sizes, shapes, and locations in cone beam CT (CBCT) images under the same constraint of memory capacity.

**Methods:**

The 2D, 2.5D, and 3D networks were compared comprehensively for the segmentation of the MS and MSL in CBCT images under the same constraint of memory capacity. MSLs were obtained by subtracting the prediction of the air region of the maxillary sinus (MSA) from that of the MS.

**Results:**

The 2.5D network showed the highest segmentation performances for the MS and MSA compared to the 2D and 3D networks. The performances of the Jaccard coefficient, Dice similarity coefficient, precision, and recall by the 2.5D network of U-net +  + reached 0.947, 0.973, 0.974, and 0.971 for the MS, respectively, and 0.787, 0.875, 0.897, and 0.858 for the MSL, respectively.

**Conclusions:**

The 2.5D segmentation network demonstrated superior segmentation performance for various MSLs with an ensemble learning approach of combining the predictions from three orthogonal planes.

## Introduction

Dental implants have become a prominent and promising treatment option for individuals with missing teeth [1]. In patients with missing maxillary teeth in the posterior region, the elevation of the maxillary sinus (MS) membrane is used to appropriately achieve the bone augmentation required for implant placement [2]. Thickening of the MS mucous membrane and pathological conditions of the MS can influence the results of bone augmentation procedures and implant treatment [3]. In the presence of mucosal thickening, determining a safe height for elevating the sinus membrane without blocking the ostium is significant [4]. According to the existing literature, most authors accepted that mucosal thickening greater than 2–3 mm is considered pathological [5]. In addition, the risk of perforation is lowest when the membrane thickness falls between 1.5-2 mm, and membranes thicker than 3 mm are more prone to perforation when MS membrane elevation is performed [6]. Sinus membrane thickening may be related to various conditions such as chronic or acute sinusitis, pseudocyst, retention cyst, mucocele, peri-apical lesions, and periodontal disease [7]. There is a significant relationship between radiographic signs of patency of sinus ostium and the mucous membrane thickness [5, 8, 9]. Therefore, accurate segmentation of the various lesions in the maxillary sinus (MSL) is essential for dental implant preoperative planning [4].

Clinicians should be cautious while planning sinus augmentation procedures in patients with radiographic evidence of sinus conditions such as mucosal thickening [4]. Cone beam CT (CBCT) has been widely used in the dental field for dental implant surgery and treatment [10, 11]. The CBCT image has the advantage of lower radiation exposure and lower cost compared to multi-detector CT (MDCT) [10, 12, 13]. However, the manual segmentation process of the MS and MSL in CBCT images is laborious and time-consuming [14, 15]. Therefore, automatic segmentation of the MS and MSL is necessary to reduce the workload of dental clinicians.

With the development of deep learning technology, there have been several recent studies on segmenting the MS and MSL using convolutional neural network (CNN) models on CBCT images [16–20]. Various segmentation networks have been proposed to automatically segment the anatomical structures from 3D medical data [21–25]. The simplest way was to sample the volume data along the orthogonal plane into 2D image sequences and train them with 2D CNN [22]. Other than 2D segmentation networks, the 3D CNN was also used to train networks for 3D information of anatomical structures from 3D volume data [23–26]. However, the 3D segmentation network required larger memory capacity compared to 2D segmentation networks [24]. To solve this problem, the 2.5D segmentation networks were proposed and applied to various medical image segmentation applications [27–34]. The 2.5D segmentation network based on 2D CNN was trained from data in axial, sagittal, and coronal planes, and the predictions from the individual 2D CNN in all three planes were ensembled [27] by unanimous [35], affirmative [35], or majority [36] voting methods. There are several different viewpoints on which training network is the best for medical image segmentation [37–40].

As far as we know, no previous studies have compared the segmentation performance of the 2D, 2.5D, and 3D networks for the segmentation of the MS and MSL. Therefore, the purpose of this study was to compare the segmentation performances of the 2D, 2.5D, and 3D networks for the MS and MSL with variations in sizes, shapes, and locations in cone beam CT (CBCT) images under the same constraint of memory capacity. Our main contributions are as follows: 1) we performed a comprehensive and quantitative comparison of 2D, 2.5D, and 3D networks for the segmentation of the MS and MSL in CBCT images under the same constraint of memory capacity and 2) the MSL was obtained by the post-processing of subtracting predictions of the maxillary sinus air region (MSA) from MS to effectively segment MSLs with large variations in sizes, shapes, and locations.

## Materials and methods

### Data acquisition and preparation

We included 67 patients (46 females and 21 males; mean age 38.18 ± 18.81 years) who underwent dental implant surgeries at the Seoul National University Dental Hospital (2020–2021). The patient data were obtained at 75 to 120 kVp and 7 to 10 mA using CBCT (DENTRI, HDX WILL Corp, Seoul, South Korea). The CBCT images had dimensions of 670 × 670 × 400 pixels, voxel sizes of 0.3 × 0.3 × 0.3 mm^3^, and 16-bit depth. This study was performed with approval from the institutional review board of the Seoul National University Dental Hospital (ERI18001). The institutional review board of Seoul National University Dental Hospital approved the waiver for the informed consent because this was a retrospective study. The study was performed in accordance with the Declaration of Helsinki.

Since it was difficult to radiologically distinguish various pathological conditions and mucosal thickening in the MS without contrast enhancement of the CBCT image, existing pathological conditions (including mucosal thickening inside the MS) were collectively regarded as the MSL in this study. MS, MSA, and MSL were manually annotated by an oral and maxillofacial radiologist using software (3D Slicer for Windows 10, Version 4.10.2; MIT, Massachusetts, USA) [41]. During the labeling process for the maxillary sinus (MS), the labels assigned to the MS did not encompass the septa within it; instead, the septa were treated as a component of the maxillary bone. In this study, our primary emphasis was on the accurate segmentation of the MS, MSA, and MSL in CBCT images.

**Fig. 1:**
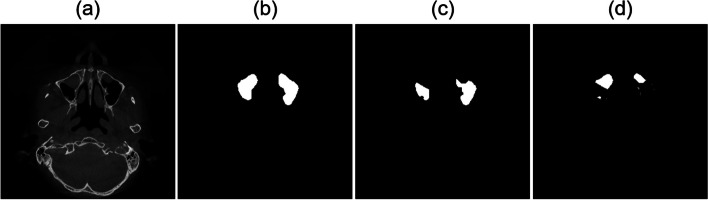
An axial slice image of a lesion inside the maxillary sinus in (**a**) CBCT images. The ground truth segmentation mask of (**b**) the maxillary sinus, (**c**) maxillary sinus air region, and (**d**) lesion inside the maxillary sinus

We estimated the minimum required sample size to detect significant differences in the accuracy among 2D, 2.5D, and 3D segmentation networks when both assessed the same subjects. Based on an effect size of 0.80, a significance level of 0.05, and a statistical power of 0.80, we acquired a sample size of *N* = 52 (G* Power for Windows 10, Version 3.1.9.7; Universität Düsseldorf, Germany). Consequently, 67 patients were divided into 53 patients and 14 patients for the training and test datasets, respectively. Within the training dataset, 39 patients were used for training and 14 for validation dataset. For a fair comparison of 2D, 2.5D, and 3D segmentation networks while meeting the memory requirement, all 3D CBCT data were resized to 256 × 256 × 192 pixels. The number of axial images used in 2D and 3D networks were 12,864 and 12,864, respectively. The number of images in 2.5D network were 12,864, 17,152, and 17,152 in axial, sagittal, and coronal planes, respectively, and they were separately used for training and ensembled afterwards. We used a multi-label segmentation approach by simultaneously segmenting the MS and MSA by deep learning, and the MSL was obtained by performing the post-processing of pixel-wise subtraction between prediction volumes of the MS and MSA to effectively segment the MSL (Fig. 1).

### 2D segmentation networks

**Fig. 2:**
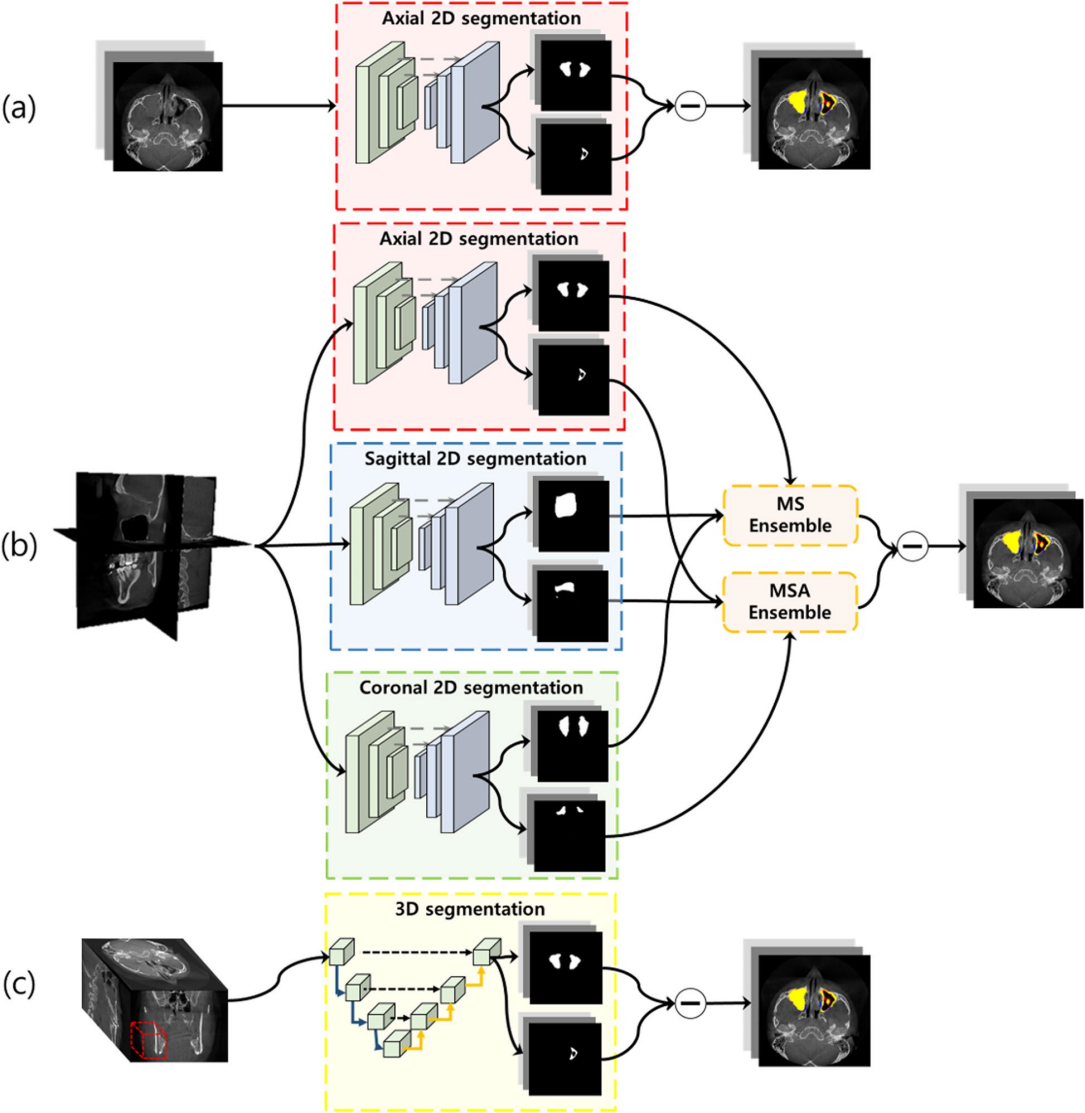
The architectures of (**a**) 2D, (**b**) 2.5D, and (**c**) 3D networks. Each network predicted the maxillary sinus and air region, and subtraction was applied to segment the lesion inside the maxillary sinus. In a 2.5D network, the same 2D network was parallelly trained with images in axial, sagittal, and coronal planes, and the predictions were ensembled before subtraction

We used deep learning networks of U-net [22] and U-net +  + [42] with backbones of ResNet101 [43], and DenseNet169 [44] for 2D segmentation (Fig. 2a). The U-net [22] architecture consisted of the encoder and decoder parts. The encoder part consisted of repeated blocks of two convolution layers followed by batch normalization, rectified linear units (ReLU), and a $2\times 2$ max-pooling for down-sampling. The decoder part, similar to the encoder, consisted of repeated blocks of up-sampling. Each block was concatenated to a corresponding feature map from the encoder block with a skip connection. The following layers were $3\times 3$ convolution, batch normalization, ReLU, and a $2\times 2$ transposed convolution for up-sampling.

The architecture of U-net +  + [42] was structurally similar to that of U-net. However, the main difference was that convolution blocks were added to the skip connections as dense skip connections instead of simply concatenating encoder and decoder feature maps with skip connections. The purpose of adding convolution blocks to skip connections was to close the gap between the feature maps of the encoder and decoder [42]. In other words, using a dense skip connection for every convolution between the encoder and decoder instead of a simple skip connection reduced the gradient vanishing problem [42].

### 2.5D segmentation networks

In 2.5D segmentation networks, the same networks were utilized as the 2D networks with backbones of ResNet101 [43] and DenseNet169 [44] (Fig. 2b). The difference was that all image data in the axial, sagittal, and coronal planes were separately used as input when training the model. The 2.5D network took a stack of continuous 2D slices in three different orthogonal planes. Each image in the three planes was used in an identical training environment as the 2D segmentation network.

The ensemble learning approach was applied to the 2.5D network for better predictive performance by combining the predictions from three orthogonal planes. The prediction results from the sagittal and coronal planes after training were transposed back to the axial plane to produce an ensemble result, which combined information from the three planes. The unanimous [35], affirmative [35], and majority [36] methods were used to generate the ensemble results from all three planes. The ensemble result was considered true if the predictions from all three planes were true by the unanimous method [35], true if one of the three predictions was true by the affirmative method [35], and true if the majority of the three predictions were true by the majority method [36].

### 3D segmentation networks

We used 3D segmentation networks for capturing 3D information (Fig. 2c). While the 2.5D networks used the same network as 2D network architectures, the 3D networks of 3D U-net [24] and 3D V-net [26] used the 3D convolution block instead of the 2D convolution block. The 3D U-net, similar to U-net, consisted of encoder and decoder structures. At the encoder part, the input was first down-sampled with 3D max-pooling with a $2\times 2\times 2$ pool size followed by repeated 3D convolution blocks of $3\times 3\times 3$ kernels and ReLU in each layer. At the decoder part, the corresponding size from the encoder was concatenated to the decoder block with a skip connection. A 3D convolution block and ReLU repeated afterward.

We also employed 3D V-net [26] for comparison with the 3D U-net. The 3D V-net architecture resembled the encoder-decoder design of 3D U-net, but it utilized an attention gate instead of skip connections between the encoder and decoder. The application of attention gates allowed the 3D V-net to extract more features compared to the basic 3D U-net. Additionally, the 3D V-net differed from the 3D U-net in the use of convolution layers instead of max-pooling layers for the down-sampling block and 3D transpose convolution layers for the up-sampling block.

### Training details for networks

To ensure a fair comparison between segmentation networks, all networks were trained using the same training setups. We designed our model as multi-task learning to simultaneously segment the background, MS, and MSA by adopting multi-label Dice loss [45]. All networks were trained using an Adam optimizer, and the learning rate of 0.001 was reduced on a plateau by a factor of 0.5 every 5 epochs over 200 epochs. The batch size was 8 for 2D and 2.5D strategies, while it was 1 for 3D networks because of computational memory limitations. Data augmentation with rotation (− 20°–20°) and brightness (− 20%–20%) was performed. The network structures were implemented with TensorFlow and Keras with NVIDIA GeForce GTX 1080 Ti 11 GB.

### Performance evaluation for segmentation

We evaluated the volume-based performances for comparisons among the prediction results by different segmentation networks. To effectively segment the MSL with large variations in sizes, shapes, and locations, we first trained the models to segment the MS and MSA, and then the MSL was obtained by performing the post-processing of pixel-wise subtraction between prediction volumes of the MS and MSA. Segmentation performances of the MS, MSA, and MSL were evaluated using the Jaccard coefficient ($JC=\frac{TP}{TP+FN+FP}$), Dice similarity coefficient ($DSC=\frac{2TP}{2TP+FN+FP}$), precision($PR=\frac{TP}{TP+FP}$), and recall ($RC=\frac{TP}{TP+FN}$) for evaluating network prediction results, where TP, FP, and FN were true positive, false positive, and false negative, respectively.

We used one-way ANOVA tests to compare performances among 2D (U-net + +), 2.5D (U-net + +), and 3D (3D U-net) networks, one-way ANOVA tests among 2D (U-net + +) networks trained with three different orthogonal planes (axial, sagittal, and coronal), one-way ANOVA tests among 2.5D (U-net + +) networks with different ensemble methods (unanimous, affirmative, and majority), and paired two-tailed t-tests between 2.5D (U-net + +) of ResNet101 [43] and Densenet169 [44] with the majority method [36] using SPSS for Windows 10 (Version 26.0, IBM, Armonk, USA). The statistical significance level was set to 0.05.

## Results

The performances of the networks were evaluated for a dataset with 14 CBCT volumes not used for training. The results in Table 1 show the segmentation performances of the JC, DSC, PR, RC, frame rate of test time (FPS), and the number of parameters (NOP) by 2D (U-net and U-net + +), 2.5D (U-net and U-net + +), and 3D (3D U-net and 3D V-net) networks. The 2.5D network showed the highest values of JC, DSC, PR, and RC for the MS, MSA, and MSL segmentation compared to other 2D and 3D networks (*p* < 0.05). The segmentation performances of JC, DSC, PR, and RC by 2.5D network (U-net + +) reached 0.947, 0.973, 0.974, and 0.971 for the MS, respectively, and 0.787, 0.875, 0.897, and 0.858 for the MSL, respectively. The 2.5D network of U-net +  + outperformed that of U-net for the MSL (*p* < 0.05). Due to the GPU memory constraint, the 3D networks resulted in lower NOP of 8.9 and 5.6 for 3D U-net and 3D V-net, respectively, however, higher FPS of 142 and 447 for 3D U-net and 3D V-net, respectively, than 2D and 2.5D networks (Table 1). As a result, the 3D networks achieved lower DSC scores with lower NOPs compared to the 2D and 2.5D networks. Therefore, the 3D network with low NOP could not sufficiently learn the structural variations in the MSA and MS, and the contrast variations of CBCT images.

**Table 1:** Segmentation performances of Jaccard coefficient (JC), Dice similarity coefficient (DSC), precision (PR), and recall (RC) for the maxillary sinus (MS), maxillary sinus air region (MSA), and the lesions inside the maxillary sinus (MSL) by 2D (U-net and U-net + +), 2.5D (U-net and U-net + +), and 3D (3D U-net, 3D V-net) networks (*: significant differences among 2D (U-net + +), 2.5D (U-net + +) and 3D (3D U-net), and †: between 2.5D (U-net) and 2.5D (U-net + +) in JC and DSC (*p* < 0.05)). The inference time was measured by frame per second (FPS), and the number of network parameters (NOP) in millions

	MSA				MS				MSL					
	JC	DSC	PR	RC	JC	DSC	PR	RC	JC	DSC	PR	RC	FPS	NOP
2D Network														
U-net	0.938 ± 0.033	0.968 ± 0.018	0.966 ± 0.015	0.970 ± 0.031	0.933 ± 0.022	0.965 ± 0.012	0.967 ± 0.015	0.963 ± 0.017	0.748 ± 0.139	0.848 ± 0.100	0.869 ± 0.080	0.832 ± 0.122	134	19.5
U-net + +	0.941 ± 0.034	0.969 ± 0.019	0.968 ± 0.014	0.970 ± 0.029	0.934 ± 0.021	0.966 ± 0.011	0.970 ± 0.013	0.961 ± 0.017	0.756 ± 0.133	0.854 ± 0.094	0.879 ± 0.074	0.835 ± 0.116	88	20.3
2.5D Network														
U-net	0.948 ± 0.030	0.973 ± 0.016	0.980 ± 0.006	0.967 ± 0.029	0.945 ± 0.019	0.972 ± 0.010	0.977 ± 0.013	0.967 ± 0.016	0.775 ± 0.132	0.866 ± 0.091	0.884 ± 0.072	0.853 ± 0.115	41	19.5
U-net + +	0.948 ± 0.030	0.973 ± 0.016	0.977 ± 0.008	0.970 ± 0.029	0.947 ± 0.018*	0.973 ± 0.010*	0.974 ± 0.013	0.971 ± 0.015	0.787 ± 0.127*†	0.875 ± 0.086*†	0.897 ± 0.053	0.858 ± 0.120	24	20.3
3D Network														
3D U-net	0.891 ± 0.058	0.941 ± 0.034	0.982 ± 0.024	0.906 ± 0.057	0.810 ± 0.109	0.891 ± 0.072	0.984 ± 0.017	0.821 ± 0.112	0.418 ± 0.240	0.545 ± 0.266	0.691 ± 0.296	0.461 ± 0.242	142	8.9
3D V-net	0.790 ± 0.098	0.879 ± 0.067	0.932 ± 0.033	0.839 ± 0.101	0.763 ± 0.099	0.862 ± 0.067	0.932 ± 0.036	0.811 ± 0.111	0.386 ± 0.227	0.515 ± 0.259	0.591 ± 0.277	0.478 ± 0.252	447	5.6

**Fig. 3:**
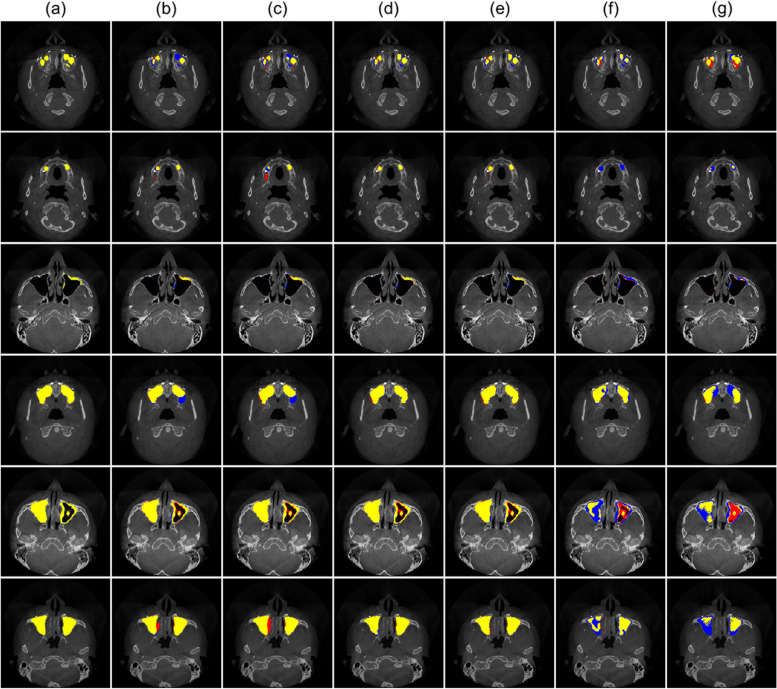
The final segmentation results of the lesion inside the maxillary sinus (MSL) by 2D, 2.5D, and 3D networks. The ground truths of the MSL (yellow) are shown in CBCT images (**a**). The false negative (blue), false positive (red), and true positive (yellow) areas are shown for the MSL segmentation by (**b**) 2D U-net, (**c**) 2D U-net +  + , (**d**) 2.5D U-net, (**e**) 2.5D U-net +  + , (**f**) 3D U-net, and (**g**) 3D V-net networks

In Fig. 3, the segmentation prediction for the various MSL by 2D, 2.5D, and 3D networks, and the ground truth were superimposed on the CBCT images. The 2.5D networks exhibited the most accurate predictions with more true positives (yellow), fewer false positives (red), and fewer false negatives (blue) compared to the other 2D and 3D networks for MSL areas of all sizes. The 2.5D network could more accurately segment the small narrow mucous membrane thickening of the MSL even just a few pixels thick, which was falsely predicted by the 3D networks. As the size of the MSL increased, there were more areas of true positive segmentation (yellow) and fewer areas of false positive (red) in 2D and 2.5D networks compared with the 3D network (Fig. 3).

**Fig. 4:**
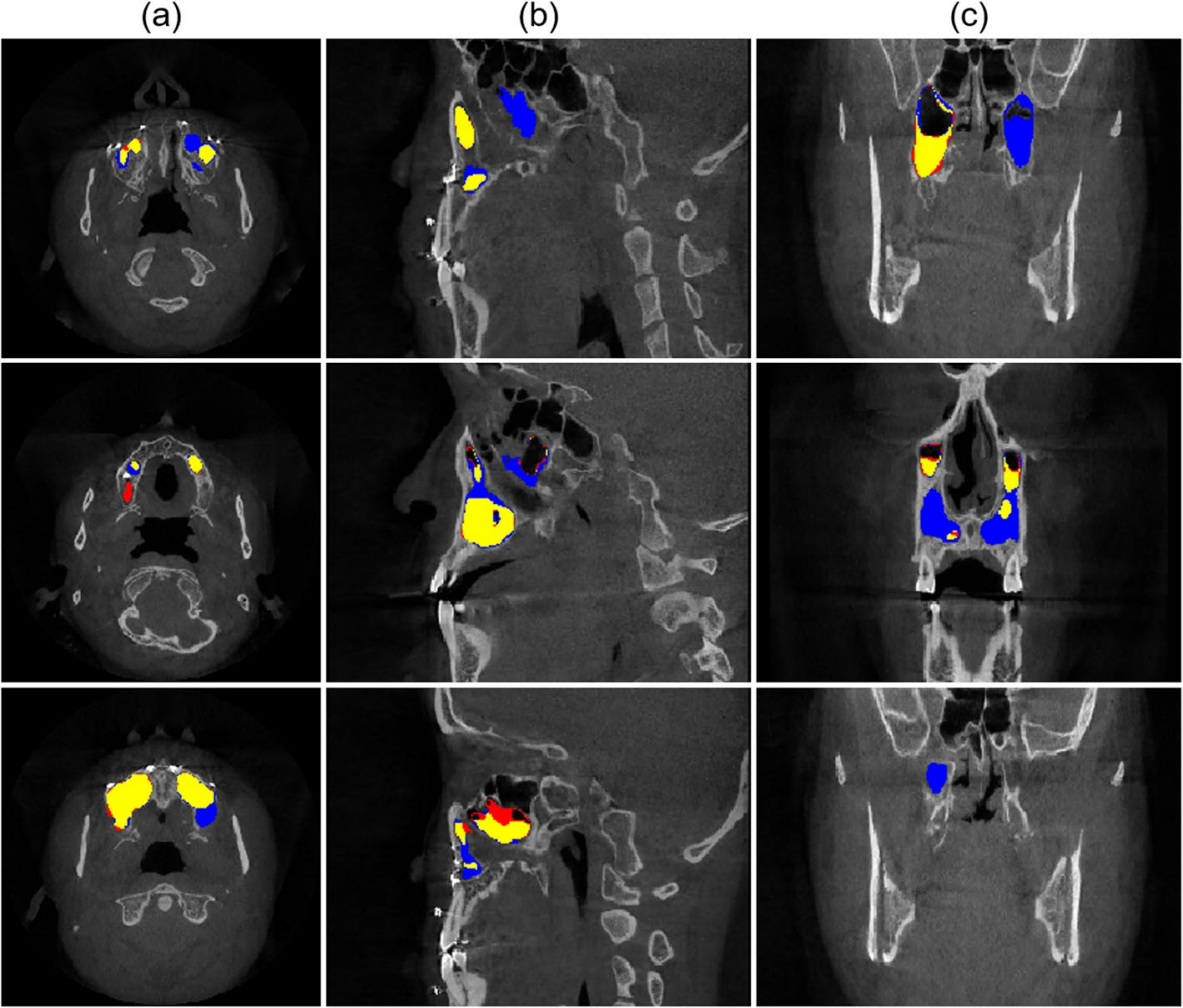
Segmentation results of the various lesions inside the maxillary sinus by 2D network in (**a**) axial, (**b**) sagittal, and (**c**) coronal planes in CBCT images. False negative (blue) and false positive (red) areas are shown in (**a**) axial, (**b**) sagittal, and (**c**) coronal planes for the lesion inside the maxillary sinus (MSL) segmentation. The true positive of the MSL segmentation is shown in yellow

**Fig. 5:**
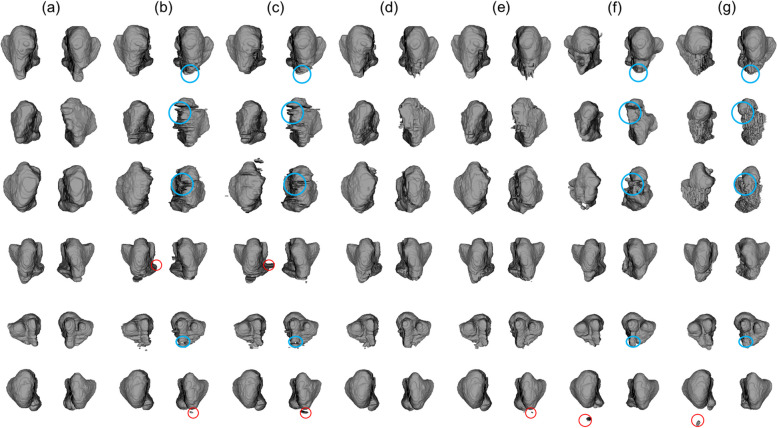
The 3D reconstruction of the maxillary sinus segmentation from (**a**) the ground truth, and by (**b**) 2D U-net, (**c**) 2D U-net +  + , (**d**) 2.5D U-net, (**e**) 2.5D U-net +  + , (**f**) 3D U-net, and (**g**) 3D V-net. The 2D and 3D networks show more false positives (red circles) than 2.5D networks, and the 2.5D networks less false negatives (blue circles) than 2D and 3D networks compared with the ground truth

**Fig. 6:**
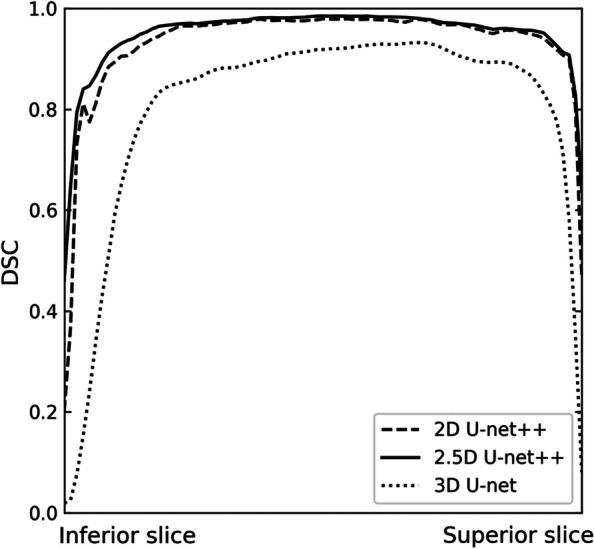
The line plots of the Dice similarity coefficient (DSC) from the inferior slice to the superior slice of the maxillary sinus by 2D U-net +  + , 2.5D U-net +  + , and 3D U-net

The 2D network showed more false negatives for the MSL in sagittal and coronal planes than in the axial plane where the MS was connected to the ethmoid sinus or the nasal cavity with ambiguous boundaries between them. Similarly, it was also difficult to segment the MSL around the floor of the MS between teeth in the axial plane (Fig. 4). In 3D results, the 2.5D U-net demonstrated better prediction with fewer false positives and false negatives in boundary details for the regions with ambiguous boundaries between sinuses and around the floor of the MS compared to the other networks (Fig. 5). As a result, the 2.5D networks generally exhibited higher performances compared to the 2D and 3D networks when the DSC for the whole volume of the MS was plotted from the inferior slice to the superior slice (Fig. 6). Therefore, the 2.5D network demonstrated the most robust performance of segmentation to the large variations in the MSL compared to the other networks.

**Fig. 7:**
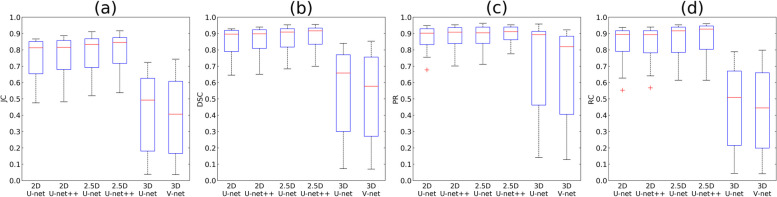
The boxplots of segmentation performance of the lesion inside the maxillary sinus for (**a**) Jaccard coefficient (JC), (**b**) Dice similarity coefficient (DSC), (**c**) precision (PR), and (**d**) recall (RC) by the 2D U-net, 2D U-net +  + , 2.5D U-net, 2.5D U-net +  + , 3D U-net, and 3D V-net. Each boxplot contains the first and third quartiles of data. The medians are located inside the boxes, visualized as red lines. The whiskers are extended above and below each box in ± 1.5 times the interquartile range (IQR), and the outliers are visualized as red + marks defining values 1.5 IQR away from the box

The segmentation performances of JC, DSC, PR, and RC for the MSL by the networks are plotted in boxplots (Fig. 7). The 2.5D networks achieved higher performances than the other networks with a smaller dispersion of data, shorter whiskers, and fewer outliers (Fig. 7). The lower segmentation performance of 3D networks for the MS and MSA compared to other networks resulted in the lowest performance for the MSL when MSA was subtracted from the MS. Therefore, the 2.5D network demonstrated the best segmentation accuracies of JC, DSC, PR, and RC for the MS and MSL among the networks.

The results in Table 2 show the segmentation performances of JC, DSC, PR, and RC by 2D (U-net + +) networks with the different backbones of ResNet101 and Densenet169, which were trained with the image data in three orthogonal planes. Some performance metrics for MS, MSA, and MSL by the 2D network trained with the axial plane showed higher values than those with the other planes (*p* < 0.05).

**Table 2:** Segmentation performances of Jaccard coefficient (JC), Dice similarity coefficient (DSC), precision (PR), and recall (RC) for the maxillary sinus (MS), maxillary sinus air region (MSA), and the lesions inside the maxillary sinus (MSL) by 2D (U-net + +) network with different backbones of ResNet101 and DenseNet169 trained with three orthogonal planes (axial, sagittal and coronal). (*: significant differences among 2D networks with axial, sagittal, and coronal planes in JC and DSC (*p* < 0.05))

	MSA				MS				MSL			
	JC	DSC	PR	RC	JC	DSC	PR	RC	JC	DSC	PR	RC
Axial												
ResNet101	0.938 ± 0.038	0.968 ± 0.021	0.972 ± 0.013	0.964 ± 0.033	0.931 ± 0.025*	0.964 ± 0.013*	0.975 ± 0.011	0.953 ± 0.021	0.742 ± 0.138*	0.844 ± 0.099*	0.883 ± 0.076	0.812 ± 0.121
DenseNet169	0.941 ± 0.034	0.969 ± 0.019	0.968 ± 0.014	0.970 ± 0.029	0.934 ± 0.021*	0.966 ± 0.011*	0.970 ± 0.013	0.961 ± 0.017	0.756 ± 0.133*	0.854 ± 0.094*	0.879 ± 0.074	0.835 ± 0.116
Sagittal												
ResNet101	0.938 ± 0.031	0.968 ± 0.017	0.972 ± 0.010	0.964 ± 0.028	0.925 ± 0.026	0.961 ± 0.014	0.969 ± 0.016	0.953 ± 0.024	0.739 ± 0.134	0.843 ± 0.096	0.881 ± 0.059	0.813 ± 0.129
DenseNet169	0.937 ± 0.030	0.967 ± 0.017	0.968 ± 0.015	0.967 ± 0.028	0.931 ± 0.024	0.964 ± 0.013	0.964 ± 0.020	0.965 ± 0.019	0.756 ± 0.126	0.855 ± 0.088	0.877 ± 0.052	0.839 ± 0.124
Coronal												
ResNet101	0.937 ± 0.031	0.967 ± 0.017	0.972 ± 0.010	0.963 ± 0.026	0.910 ± 0.050	0.952 ± 0.028	0.971 ± 0.012	0.935 ± 0.049	0.708 ± 0.150	0.819 ± 0.112	0.878 ± 0.067	0.776 ± 0.149
DenseNet169	0.933 ± 0.035	0.965 ± 0.019	0.973 ± 0.012	0.958 ± 0.031	0.930 ± 0.026	0.963 ± 0.014	0.965 ± 0.021	0.962 ± 0.018	0.747 ± 0.145	0.847 ± 0.104	0.873 ± 0.053	0.830 ± 0.148

The results in Table 3 show segmentation performances of JC, DSC, PR, and RC by 2.5D (U-net + +) networks with the backbones of ResNet101 and Densenet169 and with different ensemble methods (unanimous, affirmative, and majority). The 2.5D (U-net + +) network with the majority ensemble method achieved the highest values of the JC, DSC, PR, and RC for the MS and MSL (*p* < 0.05). The 2.5D (U-net + +) networks of DenseNet169, and the majority ensemble method showed better performance compared to ResNet101 for the MS and MSL (*p* < 0.05).

**Table 3:** Segmentation performances of Jaccard coefficient (JC), Dice similarity coefficient (DSC), precision (PR), and recall (RC) for the maxillary sinus (MS), maxillary sinus air region (MSA), and the lesions inside the maxillary sinus (MSL) by a 2.5D (U-net + +) network with different ensemble methods (unanimous, affirmative, and majority) (*: significant differences among 2.5D (U-net + +) networks with unanimous, affirmative and majority methods, and †: between 2.5D (U-net + +) of ResNet101 and Densenet169 with the majority method in JC and DSC (*p* < 0.05))

	MSA				MS				MSL			
	JC	DSC	PR	RC	JC	DSC	PR	RC	JC	DSC	PR	RC
ResNet101												
Unanimous	0.931 ± 0.041	0.964 ± 0.023	0.992 ± 0.003	0.937 ± 0.041	0.892 ± 0.062	0.942 ± 0.036	0.993 ± 0.004	0.898 ± 0.062	0.652 ± 0.170	0.775 ± 0.135	0.883 ± 0.092	0.698 ± 0.160
Affirmative	0.935 ± 0.029	0.966 ± 0.016	0.948 ± 0.020	0.985 ± 0.017	0.932 ± 0.024	0.965 ± 0.013	0.947 ± 0.022	0.984 ± 0.010	0.767 ± 0.121	0.862 ± 0.084	0.857 ± 0.062	0.871 ± 0.113
Majority	0.947 ± 0.031	0.973 ± 0.017	0.978 ± 0.007	0.968 ± 0.029	0.940 ± 0.023*	0.969 ± 0.012*	0.979 ± 0.010	0.959 ± 0.021	0.770 ± 0.132*	0.864 ± 0.091*	0.904 ± 0.054	0.831 ± 0.122
Densenet169												
Unanimous	0.931 ± 0.039	0.964 ± 0.022	0.991 ± 0.004	0.939 ± 0.039	0.921 ± 0.027	0.959 ± 0.015	0.991 ± 0.006	0.929 ± 0.028	0.715 ± 0.162	0.822 ± 0.124	0.888 ± 0.079	0.771 ± 0.151
Affirmative	0.932 ± 0.032	0.964 ± 0.018	0.943 ± 0.025	0.987 ± 0.018	0.927 ± 0.030	0.962 ± 0.017	0.938 ± 0.031	0.988 ± 0.008	0.759 ± 0.118	0.858 ± 0.082	0.847 ± 0.055	0.874 ± 0.120
Majority	0.948 ± 0.03	0.973 ± 0.016	0.977 ± 0.008	0.970 ± 0.029	0.947 ± 0.018*†	0.973 ± 0.010*†	0.974 ± 0.013	0.971 ± 0.015	0.787 ± 0.127*†	0.875 ± 0.086*†	0.897 ± 0.053	0.858 ± 0.120

## Discussion

CBCT images are extensively used for dental implant surgical planning in the field of dentistry [10], and they offer several advantages, including reduced radiation exposure and lower cost compared to multi-detector CT [10]. The accurate segmentation of MS and MSL in CBCT images enables dental clinicians to precisely visualize the size, shape, and location of the MSL. The 3D segmentation information of the MSL is essential for determining the appropriate treatment approach [4]. However, the manual segmentation process of the MS and MSL in CBCT images is laborious and time-consuming [14, 15]. Therefore, automatic segmentation methods were required to alleviate the workload of dental clinicians.

Advancements in deep learning led to the development of various deep learning models designed for the automatic segmentation of the MS and MSL in CBCT images [16–20]. Morgan et al. [16], Nogueira-Reis et al. [18], and Choi et al. [19] showed DSC values of 0.996, 0.984, and 0.910, respectively for the MS using 2D or 3D networks, and Jung et al. [17] showed DSC values of 0.930, 0.760 for the MSA and MSL in CBCT images using a 3D network. Hung et al. [20] showed DSC values of 0.972, 0.729, and 0.678 for the MSA, mucosal thickening and mucosal retention cysts, respectively, in CBCT images by 3D network. These 2D or 3D networks provided efficient and accurate segmentation results of the MS, MSA, and MSL in CBCT images, which could be alternatives to manual segmentation. Although previous studies performed the MS, MSA, and MSL segmentation in CBCT or CT images using 2D or 3D networks, it was unclear which network (2D or 3D) was best in terms of segmentation performance. Therefore, we compared the segmentation performances of the MS and MSL by the different 2D, 2.5D, and 3D networks with various backbones and ensemble methods. As far as we know, no previous studies have been performed to compare the segmentation performance of the MS and MSL among 2D, 2.5D, and 3D networks.

The limited size of the MSL dataset presented challenges for the deep learning model to effectively learn contextual information of the MSL. The varying sizes, shapes, and locations of the MSL, which only occupied a small portion of the sinus, made it difficult for the model to generalize from a limited dataset [4]. As a result, the model encountered difficulties in accurately identifying and delineating the anatomical structure of the MSL due to the insufficient training dataset. To address this issue, we adopted an alternative approach to segmenting the MSL, where we performed an indirect segmentation method by subtraction between prediction volumes of the MS and MSA. This involved pixel-wise subtraction of the segmentation volume of the MSA from the MS prediction. By utilizing the subtraction approach between the MSA and MSL predicted by the model, we were able to obtain a more accurate segmentation of the various MSLs in CBCT images.

The segmentation of the MS and the MSL by the 2.5D network exhibited superior performance in terms of JC, DSC, PR, and RC compared to 2D and 3D networks. Although the performance of the 2D network was comparable with the 2.5D network in segmenting the MS and MSA, there was a significant difference in MSL segmentation. In the MSL of small narrow mucous membrane thickening (even when it is just a few pixels thick), false predictions in the segmentation of the MS and the MSA could result in false segmentation of the entire MSL. Like 2D networks, 2.5D networks were trained using 2D slice images acquired from three orthogonal planes (i.e., axial, sagittal, and coronal). Despite working with only the 2D slice images, the 2.5D networks were able to leverage the 3D information inherent in combining the multiple planes by ensemble methods. This enabled more accurate segmentation results compared to conventional 2D networks with the advantage of requiring only the memory capacity of a 2D network [27].

The 2D network was trained with 2D slices from three orthogonal planes (axial, sagittal, coronal). Generally, the result trained with the axial plane outperformed some evaluation metrics compared to the two other planes. Although the 2D networks effectively segmented the MS and MSA, they had some limitations in capturing the 3D information of the MS and MSL by only learning the 2D information of each plane in CBCT images. The 2D network showed more false negatives for the MS in the sagittal and coronal planes than in the axial plane when the MS was connected to the ethmoid sinus or the nasal cavity with ambiguous boundaries between them. Similarly, it was more difficult to segment the MS around the floor of the MS between teeth in the axial plane. As a result, an MSL with large variations in size, shape, and location of the MS was more visible in a specific plane than in others. Therefore, a 2.5D network ensemble of the predictions in the axial, sagittal, and coronal planes resulted in improvements in segmentation performance compared to 2D networks.

We compared different ensemble methods (unanimous, affirmative, and majority) for the 2.5D network (U-net + +) with two backbones. The ensemble method of the majority voting for predictions in the axial, sagittal, and coronal planes was found to be more effective in improving the performance of the MSL segmentation in CBCT images. The affirmative method achieved the highest RC value for the MSL segmentation, indicating that this method was more effective in reducing the false negatives. However, there was a decrease in the PR value, showing that it increased the false positives and resulted in a lower DSC value compared to the majority method. If the segmentation predictions in sagittal or coronal planes had lower false positives, then the affirmative method could result in a similar performance to the majority method.

We observed that 3D networks were not always better than 2D or 2.5D networks in segmentation of the MS and MSL under the same constraint of GPU memory capacity. These results were attributed to two main reasons. First, the reduced number of parameters of 3D networks was used due to the GPU memory constraints [46]. The 3D networks with the reduced number of parameters could negatively affect 3D networks in learning contextual information sufficiently about the different sizes, shapes, and locations of the MSLs (Table 1). Second, as the data augmentation for 3D networks had limited variation in the dataset than 2D networks by the GPU memory constraints, the 3D network did not sufficiently learn the structural variations in the MSA and MS and the contrast variations of CBCT images. Specifically, even though the 3D network was capable of capturing more 3D information than the 2D network, the 3D networks weren’t able to learn enough 3D anatomical variation to achieve better performance, due to an insufficient number of data and model parameters. The 3D network had more false negatives, particularly in the posterior region where there was substantial variation in the shapes of the MS and MSL across patients (Fig. 5). As a result, the 3D networks were more likely to overfit the training set compared to the 2D networks [27, 29].

The major finding in this study was that 2.5D networks resulted in more effective and accurate segmentation of the MSL by subtraction between predictions of the MS and MSA than 2D and 3D networks under the same constraint of GPU memory capacity. Nevertheless, there were several limitations in this study. First, we only used an internal dataset from a single organization to train deep learning models, which resulted in a potential limitation of generalization. The performance of the 2D, 2.5D, and 3D networks might have different results by changing the dataset with external data. The networks need to be trained and evaluated using large datasets from multiple organizations or devices for generalization. Second, further research is needed to investigate whether the findings of this study also remain consistent when applying different network architectures such as transformer [47], UNETR [48], Swin Transformer [49], and SegFormer [50] for segmentation of the MS, MSA, and MSL in CBCT images.

## Conclusions

In this study, we compared the segmentation performance of the MS and MSL in CBCT images using 2D, 2.5D, and 3D networks under the same constraint of memory capacity. The 2.5D network demonstrated superior performance for segmentation of the various MSL with the ensemble learning approach of combining the predictions from three orthogonal planes. Furthermore, the networks could effectively segment the various MSL by subtraction between predictions of the MS and MSA. The 2.5D network contributed to a more accurate evaluation of both the MS and MSL structures by improving robustness to structural variations and providing details on anatomical boundaries in CBCT images for the preoperative planning of implant surgeries to minimize surgical complications.

## Data Availability

The datasets generated and/or analyzed during the current study are not publicly available due to the restriction by the IRB of Seoul National University Dental Hospital to protect patient privacy but are available from the corresponding author on reasonable request. Please contact the corresponding author for any commercial implementation of our research.
